# Simulation and Experimental Study of Silica-Based Adsorbents for Competitive Adsorption of Toluene from Binary Components

**DOI:** 10.3390/ma19143097

**Published:** 2026-07-19

**Authors:** Yuxin Shi, Shengzhuo Yuan, Shiyu Hou, Gen Huang, Wanci Shen, Feiyu Kang, Zheng-Hong Huang

**Affiliations:** 1 School of Chemical and Environmental Engineering, China University of Mining and Technology (Beijing), Beijing 100083, China; xinxin8640@163.com (Y.S.); ysz1565322666@163.com (S.Y.); 2 Key Laboratory of Advanced Materials (MOE), State Key Laboratory of New Ceramics and Fine Processing, School of Materials Science and Engineering, Tsinghua University, Beijing 100084, China; shenwc@mail.tsinghua.edu.cn; 3 Tsinghua Shenzhen International Graduate School, Tsinghua University, Shenzhen 518055, China; fykang@tsinghua.edu.cn

**Keywords:** molecular dynamics simulation, adsorption, pore size, structure-activity relationship, selective

## Abstract

**Highlights:**

- MD simulation and IAST were employed to predict the selectivity of toluene.
- Pore size plays a pivotal role in achieving the selective separation of toluene.
- Adsorbents with a pore size of 0.7 nm show the highest selectivity for toluene.
- The experimental results are basically consistent with the simulation results.

**Abstract:**

Physical adsorption is a green separation technology with industrial potential. Precise control of the pore structure of adsorbent is key to achieving highly selective separation of target molecules of similar size. In this study, a combined molecular simulation and experimental approach was employed to investigate the influence of pore size on the adsorption behavior of benzene, toluene and o-xylene over silica-based adsorbents. The simulation results show that methyl groups enhance the interfacial interactions between adsorbate molecules and pore walls, whereas steric hindrance limits the packing efficiency of the adsorbates within the pores. Consequently, adsorption selectivity is governed by the balance between confinement-enhanced adsorbate–adsorbent interactions and steric accessibility, with a pore size of approximately 0.7 nm providing the most favorable adsorption environment for toluene. The experimental results are consistent with the simulation results. When benzene and toluene are co-adsorbed, the NaY adsorbent with an average pore size of 0.7 nm exhibits the highest adsorption capacity and selectivity for toluene.

## 1. Introduction

Toluene is a critical aromatic hydrocarbon chemical feedstock, widely employed in diverse industrial sectors including coatings [1], resins [2] and pharmaceutical fine chemical synthesis. It also acts as a key component in solvent oils and fuel blends [3], while serving as an essential precursor for derivatives such as styrene and benzoic acid [4,5]. Consequently, the procurement of high-purity toluene remains indispensable for downstream product integrity and sustained operational stability [6]. In current industrial practice, toluene separation and purification are dominated by conventional distillation techniques, which fundamentally hinge on the volatility discrepancies between toluene and its impurities to drive the fractionation process [7]. However, in actual industrial production, toluene coexists with benzene, o-xylene and other aromatic hydrocarbons to form azeotropic or near-azeotropic mixtures. This leads to highly convergent vapor-liquid equilibrium curves, significantly increasing the complexity of separation [8]. In addition, industrial distillation processes often demand extremely high reflux ratios, multi-stage packed columns in series, and complex coupled tower system designs [9]. Meanwhile, most energy consumed during distillation is derived from fossil fuels, indirectly contributing to significant carbon dioxide emissions [10]. For the recovery of benzene and toluene, the extraction distillation process achieves recovery rates of 99.9% for both compounds by organically integrating extraction distillation with liquid–liquid extraction. However, the overall separation process remains reliant on high-energy thermal inputs [11]. Against the dual backdrop of rising energy costs and low-carbon demands, developing alternative separation technologies that are low-temperature, low-energy, and highly selective has emerged as a key research focus in the aromatics industry.

Adsorption-based separation has emerged as a promising alternative to conventional distillation processes because of its low energy consumption, mild operating conditions, and facile regeneration characteristics [12]. The selective separation of mixed aromatic hydrocarbons via adsorption is primarily determined by the interplay between adsorption affinity and molecular diffusion behavior within porous adsorbents. Differences in molecular size, geometry, and polarity give rise to distinct diffusion resistances, adsorption configurations, and spatial compatibility with active sites, which collectively lead to divergent adsorption performances [13]. The differences in size, structure and polarity of adsorbate molecules result in distinct variations in diffusion resistance within adsorbents with specific pore sizes and topologies, as well as in the spatial matching degree with adsorption sites [14]. Consequently, pore size distribution, pore geometry, and framework topology are widely recognized as the key structural parameters governing adsorption selectivity in porous materials [15]. Silica-based porous adsorbents, including zeolites and ordered mesoporous silica, are ideal model materials for investigating pore-size-dependent adsorption phenomena because of their well-defined pore structures, excellent thermal stability, and tunable framework architecture [16]. In particular, zeolitic materials possess highly uniform microporous channels that are comparable to the kinetic diameters of aromatic hydrocarbons, enabling molecular sieving effects and selective adsorption behavior [17]. Molecular sieve materials exhibit significant advantages in the selective adsorption of aromatic compounds due to their highly ordered pore structures, extremely narrow pore size distributions, and excellent thermal stability [18]. Previous studies have demonstrated that pore structure exerts a profound influence on the adsorption of benzene, toluene, ethylbenzene, and xylene (BTEX) molecules. The pore size and topological characteristics of molecular sieves closely match the kinetic diameters of aromatic molecules, representing a key structural factor governing adsorption selectivity [19]. Guo et al. [20] reported that adsorption capacity and selectivity are strongly correlated with pore architecture and pore size distribution. Furthermore, molecular simulations by Shen et al. [21] demonstrated that in non-polar aromatic systems, molecular sieves exhibit a decreasing adsorption affinity for substances with increasing molecular volume, providing robust theoretical support for leveraging pore structure to achieve molecular sieve effects. Wu et al. [22] investigated competitive adsorption experiments involving six aliphatic volatile organic compounds (VOCs) through dynamic experiments and molecular simulations, indicating that highly polar VOCs exhibit greater competitive advantage. Moreover, Altaf et al. [23] found that pore sizes in the range of 0.59–1.50 nm exhibited superior adsorption performance for aromatic VOCs, highlighting the importance of pore-size optimization. Recent studies on carbon molecular sieves have also revealed that subtle regulation of sub-nanometer pore dimensions can dramatically enhance molecular screening among similarly sized adsorbates [24].

Due to subtle differences in molecular size and spatial configuration among adsorbate molecules, when the pore size of the molecular sieve is comparable to their kinetic diameters, the disparities in diffusion resistance and the extent of configurational restriction within the pores are significantly amplified Therefore, from a kinetic perspective, molecules tend to occupy adsorption sites based on “pore accessibility” rather than solely on the order of adsorption affinity strength, making pore structure the dominant factor governing adsorption priority and selectivity in multicomponent systems [25]. Additionally, components with strong adsorption affinity or high boiling points tend to preferentially occupy favorable adsorption sites. Under certain conditions, these components may even induce adsorption site displacement, thereby significantly altering the adsorption performance of the target component [26]. Zhou et al. [27] successfully prepared a carbon molecular sieve with a pore size of 0.44 nm, which precisely separated C3H6 from C3H8 with an adsorption ratio reaching 145.4. Altaf et al. [23] demonstrated that adsorption materials with pore sizes ranging from 0.59 to 1.50 nm exhibit a stronger correlation with the dynamic adsorption capacity of toluene. Hu et al. [28] found that the pore size distribution of the material in the range of 1.7–3.0 nm is suitable for adsorbing BTEX molecules. This is because it matches well with the molecular diameters of BTEX molecules (benzene, toluene, ethylbenzene, xylene, etc.), which are 0.59–0.74 nm. Therefore, selective enrichment of target components can be achieved by precisely regulating the pore size and channel topology of adsorbents. However, there is a lack of adsorbent screening and mechanism elucidation specifically oriented towards the toluene purification process. Notably, molecular dynamics (MD) simulation methods can construct idealized models to reveal the intrinsic mechanisms by which pore size and topological structure influence adsorption selectivity, thereby guiding adsorbent design at the molecular level.

In this work, silica-based adsorbent models with different pore sizes were constructed to reveal the structure-activity relationship between pore size and adsorbate molecules at the molecular scale. The adsorption isotherms of benzene, toluene and o-xylene were simulated, and the adsorption selectivity of the different models for toluene was evaluated by the Ideal Adsorption Solution Theory (IAST). In addition, three commercial silica-based adsorbents (NaY, 13X and MCM-41) were selected to conduct static adsorption experiments, single-component dynamic adsorption experiments and binary-component competitive adsorption experiments to verify the simulation results. This study quantitatively determined that approximately 0.7 nm is the optimal pore size for maximizing toluene selectivity and revealed the competitive interaction between adsorption affinity and steric hindrance. This validated framework provides concrete design guidelines for pore size optimization and offers theoretical support for the deep purification of toluene.

## 2. Materials and Methods

### 2.1. Molecular Dynamics Simulation

To develop the adsorption simulation, structure models of the adsorbents with pore sizes of 0.5 nm, 0.7 nm, 1.0 nm, 1.2 nm, 1.4 nm and 2.0 nm were first constructed with the software Material Studio (MS) (Figure S1). Then, MD calculations were performed using the GROMACS 2018 program package [29]. Data analysis and visualization were performed using the VMD software [30]. The simulation details are described in the Supplementary Materials.

### 2.2. Materials and Reagents

13X was procured from Shanghai Acmec Biochemical Co., Ltd. (Shanghai, China), while NaY and MCM-41 were obtained from Jiangsu XFNANO Materials Tech Co., Ltd. (Nanjing, China). A benzene, toluene, and o-xylene standard gas with a concentration of 1000 ppm was supplied by Beijing Haipu North Branch Gas Industry Co., Ltd. (Beijing, China). Benzene (assay ≥ 99.7%) and toluene (analytical reagent) were purchased from Aladdin Company (Shanghai, China), and o-xylene was supplied by Anhui Zesheng Technology Co., Ltd. (Shanghai, China).

### 2.3. Materials Characterization

The pore structure of adsorbents was determined via nitrogen adsorption–desorption isotherms at a temperature of 77 K on a physical adsorption instrument (BELFOR-II, MicrotracBEL, Osaka, Japan). Prior to each test, adsorbents underwent vacuum degassing at 473 K for 6 h to eliminate impurities and water. The specific surface area of the adsorbents was determined using the Brunauer–Emmett–Teller (BET) method. For pore size distribution analysis, NaY was fitted using density functional theory (DFT), 13X using SF, and MCM-41 using the BJH method. The total pore volume was estimated from the amount of nitrogen adsorbed at a relative pressure (P/P_0_) of about 0.95. Fourier Transform Infrared Spectroscopy (FTIR) characterizations were performed on a Nicolet iS20 instrument (Thermo Fisher Scientific, Waltham, MA, USA) to analyze the surface functional groups of the adsorbents; the spectra were recorded with a resolution of 4 cm^−1^ in the range of 400–4000 cm^−1^. The crystal structure of the adsorbents was determined by X-ray diffraction (XRD, Rigaku Corporation, Tokyo, Japan) with Cu-Kα radiation at a pipe pressure of 40 kV; the wavelength (λ) was 1.54056 Å. The XRD patterns of NaY and 13X were collected over a 2θ range of 5° to 90° at a scanning rate of 5°·min^−1^, while that of MCM-41 was collected over a range of 0.5–10° and with a scanning rate of 0.5°·min^−1^.

### 2.4. Evaluation of Adsorption Performance

The adsorption isotherms of benzene, toluene and o-xylene at 303 K were determined using a Physical adsorption instrument (BSD-660MV, Beishide Instrument Technology (Beijing) Co., Ltd., Beijing, China). All samples were pretreated for 6 h at 473 K within a vacuum heating system before testing. The dynamic adsorption performances of benzene, toluene and o-xylene were evaluated through fixed-bed adsorption experiments (Figure S2). Then 0.2 g of the adsorbent with a particle size of 40–60 meshwas placed into an adsorption tube with a diameter of 6 mm and pretreated with a nitrogen gas flow of 100 mL·min^−1^ at 473 K for 2 h before the test to remove impurities. In the experiments of single-component adsorption and binary-component co-adsorption, the concentrations of benzene, toluene and o-xylene were all 1000 ppm, and the environmental temperature was 303 K. The total gas flow through the adsorption tube was 100 mL·min^−1^, with a gas hourly space velocity (GHSV) of 8850 h^−1^. Benzene, toluene and o-xylene concentrations at the reactor inlet and outlet were carried out by a gas chromatograph (GC-2014, Shimadzu, Kyoto, Japan) equipped with a flame ionization detector (FID). All adsorption experiments were conducted independently, with each set of experiments repeated three times; data are presented as mean ± standard deviation (SD). The total adsorption capacity of the target component of the adsorbents was calculated by the following formula:

(1) $$Q=\frac{FA\int (Cin-Cout)dt}{10^{6}\cdot W}$$

where Q is the adsorption capacity (mg·g^−1^), F_A_ is the gas flow rate (mL·min^−1^), W is the mass of the adsorbent (g), and C_out_ and *C*_in_ are the concentrations of the adsorbate molecules at the outlet and inlet, respectively (mg·m^−3^).

The competitive adsorption coefficient in the adsorbent was calculated using the following formula:

(2) $${Competitive\;coefficient}_{i/j}=\frac{{\left(S_{i}-B_{i}\right)/S}_{i}}{{\left(S_{j}-B_{j}\right)/S}_{j}}$$

where competitive coefficient i/j is the competitive coefficient of component i for component j in binary-component co-adsorption, dimensionless; Si and Bi are the adsorption capacity of component i in single-component adsorption and binary-component co-adsorption, respectively, mg/g; Sj and Bj are the adsorption capacity of component j in single-component adsorption and binary-component co-adsorption, respectively, mg/g.

## 3. Results and Discussion

### 3.1. Simulation of Adsorption with Silica-Based Adsorbent Models for Benzene, Toluene and O-Xylene

To elucidate the influence of pore architecture on adsorption behavior, silica-based porous models with pore sizes ranging from 0.5 to 2.0 nm were constructed, and the adsorption properties toward benzene, toluene, and o-xylene were investigated by molecular simulations. To investigate the influence of concentration (partial pressure) on the adsorption process, adsorption isotherms under different relative pressures (P/P_0_ = 1.0 and 0.1, where P0 is the saturated vapor pressure of benzene, toluene and o-xylene at 303 K, respectively) were simulated. Figure S3 depicts the adsorption isotherms of P/P_0_ over the range from 0 to 1. It is observed that benzene, toluene, and o-xylene predominantly exhibit type I adsorption isotherms across different pore-size adsorbent models, indicative of the characteristic pore-filling adsorption behavior. Notably, benzene exhibits the highest adsorption capacity in the model, with a pore size of 1.2 nm, whereas toluene and o-xylene achieve their maximum adsorption capacity in the 2.0 nm model. This trend arises because the optimal pore size for adsorption increases with the molecular size of the adsorbate [31]. Additionally, the steric hindrance effect of adsorbate molecules within the pore channels further limits the filling amount of large-sized adsorbate molecules in narrow-pore models.

**Figure 1:**
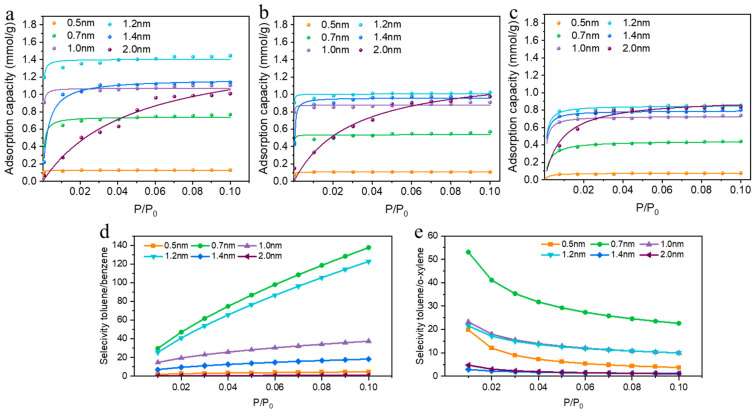
Simulated adsorption isotherms of (**a**) benzene, (**b**) toluene and (**c**) o-xylene on adsorbent models at P/P_0_ = 0–0.1 (P_0_ is the saturated vapor pressure of benzene, toluene and o-xylene at 303 K, respectively). Predicted selectivity of (**d**) toluene/benzene and (**e**) toluene/o-xylene (At 303 K, the mixture was studied on the adsorbent model with the adsorbates in a 1:1 molar ratio).

Figure 1 further simulates the adsorption behaviors of real concentrations of benzene, toluene and o-xylene (the adsorption isotherm under the condition of P/P_0_ = 0–0.1). The adsorption behaviors of benzene, toluene and o-xylene within adsorbent models with different pore sizes were systematically investigated. The simulation results indicate that the adsorption capacity first increases and then decreases with the increase in pore size, which suggests that there is an optimal range of pore size for the adsorption of adsorbates with different molecular diameters. In addition, except for the 2.0 nm pore-size adsorbent model, the adsorption capacity of all adsorbate molecules increases rapidly in the low partial pressure range and reaches a plateau in adsorbent models with pore size ranges from 0.5 nm to 1.4 nm. This indicates that the adsorption process is primarily governed by a micropore filling mechanism, where a strong interaction occurs when the size of the adsorbate molecule is comparable to that of the adsorbent pore size. The adsorption capacity of the adsorbate molecules in the adsorbent model with a pore size of 2.0 nm gradually increases with the increase in partial pressure, demonstrating the characteristic of multi-layer molecular adsorption. Furthermore, within the same pore-size adsorbent model, the adsorption capacity follows the order: benzene > toluene > o-xylene. The adsorption capacity of benzene, toluene and o-xylene on the adsorbent model with a pore size of 1.2 nm is 1.4 mmol/g, 1.0 mmol/g and 0.8 mmol/g, respectively. This trend is attributed to the steric hindrance effect, which limits the ability of larger molecules to fill the pores effectively. Within the same adsorbent model, adsorption capacity followed the order benzene > toluene > o-xylene. This trend originated from the combined effects of steric hindrance and configurational restriction under confined pore volume. As molecular size increased from benzene to o-xylene, diffusion resistance and packing restriction within narrow pore channels became increasingly significant, thereby limiting pore filling efficiency.

The selectivity coefficient is a critical parameter for evaluating the competitive adsorption ability of adsorbate molecules in binary-component co-adsorption systems [32]. Figure 1d presents the selectivity coefficients of adsorbent models with different pore sizes for toluene and benzene. It is observed that, except for the 0.5 nm pore-size model, the toluene/benzene selectivity coefficient consistently exceeds 1. This is because toluene contains an additional methyl group compared to benzene, which strengthens the interaction between toluene and the adsorbent pore channels. Due to the steric hindrance effect of large adsorbate molecules in narrow pores, adsorbent models with pore sizes of 0.5–1.2 nm exhibit higher adsorption selectivity for toluene than for o-xylene (Figure 1e). This is because the two adjacent methyl groups in o-xylene induce significant steric hindrance, and its larger kinetic diameter (0.71 nm for o-xylene compared to 0.61 nm for toluene) leads to a higher diffusion energy barrier when entering the pore channels [28]. Furthermore, the toluene/benzene selectivity coefficient increases with increasing partial pressure, whereas the toluene/o-xylene selectivity coefficient decreases with increasing partial pressure. This phenomenon occurs because high partial pressure enhances the mass transfer driving force of adsorbate molecules during adsorption and mitigates the steric hindrance effect. Notably, the 0.7 nm pore-size adsorbent model demonstrates the highest adsorption selectivity for toluene. This behavior can be attributed to an optimal pore-size matching effect between the microporous channels and the molecular dimensions of toluene, which enhances adsorption affinity while maintaining sufficient diffusion accessibility. In contrast, larger pore sizes mainly provide increased free volume but weaker confinement effects, resulting in reduced discrimination among aromatic molecules with similar structures. The results indicate that the adsorption selectivity is governed by the balance between confinement-enhanced adsorption interactions and steric accessibility. Since the adsorption of benzene, toluene, and o-xylene is generally governed by non-covalent interactions rather than the formation of new chemical bonds, the proposed mechanism is primarily manifested in differences in molecular packing and adsorption affinity within the pore channels. These findings suggest that pore-size matching provides an effective structural strategy for regulating the selective adsorption of aromatic hydrocarbons [33,34].

### 3.2. Characterization of Adsorbents

**Figure 2:**
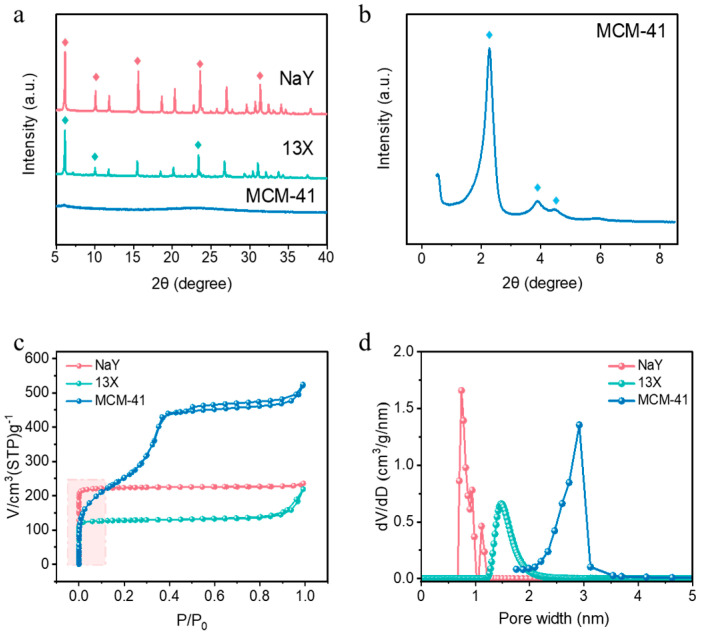
XRD patterns of (**a**) NaY and 13X; (**b**) small-angle X-ray diffraction of MCM-41; (**c**) nitrogen adsorption isotherms of NaY, 13X and MCM-41 at 77 K.(The red box highlights the low-pressure region (P/P_0_ < 0.1)); (**d**) pore size distribution of NaY, 13X and MCM-41.

Three silica-based materials with distinct pore structures were selected for experimental verification, including two microporous materials, NaY and 13X, and the mesoporous material MCM-41. As shown in Figure 2a, both NaY and 13X exhibit the characteristic diffraction peaks of the FAU-type zeolite framework. The diffraction peaks located at approximately 2θ = 6.2°, 10.2°, 11.9°, 15.7°, 20.3°, 23.7°, 27.1° and 31.4° can be indexed to the (111), (220), (311), (331), (511), (440), (533) and (642) crystal planes of the FAU structure, respectively, which are in good agreement with the standard diffraction pattern of FAU-type zeolites (JCPDS 43-0168), confirming the successful formation of highly crystalline FAU aluminosilicate zeolites [31,32,35]. Figure 2b shows MCM-41 displaying prominent characteristic peaks at 2θ = 2.27°, 3.8° and 4.4°, corresponding to the (100), (110) and (200) crystal planes [36,37]. It indicates the material possesses a highly ordered two-dimensional hexagonal mesoporous structure (p6m) [38], and the spacing of the (100) crystal plane (d100) is 3.90 nm according to the Bragg formula [39]. Figure 2c shows the nitrogen adsorption–desorption curves for NaY, 13X and MCM-41. The results indicate that the curves of NaY and 13X exhibit type-I adsorption isotherms, with adsorption capacity significantly increasing at low relative pressures, suggesting the presence of abundant micropores within the adsorbents. In contrast, the curve of MCM-41 displays a typical type-IV adsorption isotherm with an H1 hysteresis loop, demonstrating a pronounced cylindrical mesoporous structure. Figure 2d presents the pore size distribution curves of three adsorbents, and the pore structure parameters are listed in Table S1. All three adsorbents have a narrow pore size distribution, with average pore sizes of 0.7 nm, 1.0 nm and 2.2 nm for NaY, 13X and MCM-41, respectively. The experimental pore dimensions are consistent with those employed in molecular simulations, enabling direct validation of the predicted pore-size-dependent adsorption behavior.

Figure S4 presents the FTIR spectra of the three adsorbents. The peak at 660 cm^−1^ is assigned to the bending vibration of siloxane bonds, while the peak at 1230 cm^−1^ corresponds to the symmetric stretching vibration of siloxane bonds. Peaks at approximately 480 cm^−1^, 550 cm^−1^, 740 cm^−1^ and 982 cm^−1^ are attributed to Si-O-Al bending vibrations [40]. The intense absorption peak at 1005 cm^−1^ is associated with the asymmetric stretching vibration of the Si-O-Si bond, which forms the primary framework of MCM-41 [41,42]. Additionally, the broad absorption band at 3480 cm^−1^ is assigned to surface hydroxyl groups [40,43]. These characterization results indicate that the three adsorbents exhibit no significant differences in surface oxygen-containing functional groups, and surface properties have a relatively minor impact on adsorption performance.

### 3.3. Effect of Pore Architecture on Static Adsorption Behavior

**Figure 3:**
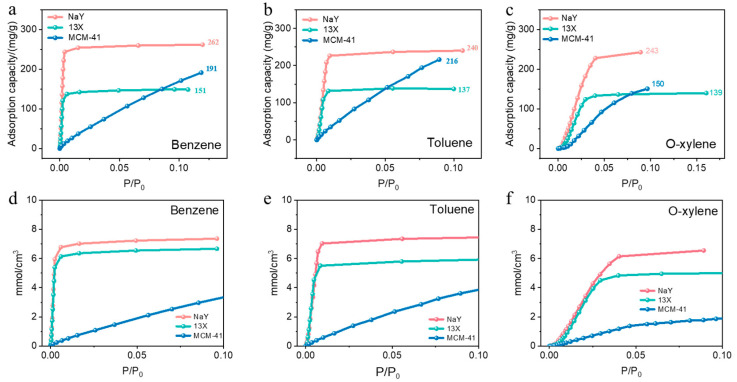
Adsorption isotherms of NaY, 13X and MCM-41 for (**a**) benzene, (**b**) toluene and (**c**) o-xylene at 303 K; adsorption amounts of (**d**) benzene, (**e**) toluene and (**f**) o-xylene per unit pore volume of NaY, 13X and MCM-41.

NaY, 13X, and MCM-41 not only have different pore structures but also differ in their framework compositions, Si/Al ratios, and cation distributions. These characteristics may influence the adsorption strength of aromatic hydrocarbons to some extent through differences in framework polarity and interactions between the adsorbate and the framework. However, this study focuses primarily on the effects of pore structure and pore-size matching on adsorption behavior. Static adsorption experiments of benzene, toluene, and o-xylene on NaY, 13X, and MCM-41 were conducted to further reveal the influence of pore structure on adsorption behavior. Figure 3a,b show the adsorption isotherms of NaY and 13X for three adsorbate molecules, which exhibit typical type-I characteristics. The full-range isotherms (P/P0 = 0–1) are presented in Figure S5, which confirm the type-I behavior and the saturation plateau observed at higher relative pressures. Their adsorption capacities rise sharply at extremely low pressures (P/P_0_ < 0.01) and rapidly reach a saturation plateau, indicating rapid filling of micropores at very low relative pressures. MCM-41 shows typical IV-type isotherm characteristics (Figure 3c), and the adsorption amounts of benzene and toluene increase sharply in the high-pressure zone due to capillary condensation. In the static adsorption experiment, the changes in adsorption isotherms of benzene, toluene and o-xylene by different pore-sized adsorbents were consistent with the simulation results. To eliminate the influence of pore volume variations and evaluate pore utilization efficiency, adsorption capacity was normalized per unit pore volume of adsorbents. Figure 3d–f demonstrate that NaY exhibits the highest adsorption capacity for all target adsorbates, which contradicts the simulation results. This is probably because sodium atoms in NaY could form π-bond interactions with benzene rings, thereby enhancing the interaction strength between the pores and the adsorbate molecules [44]. The filling densities of all adsorbents for adsorbates follow the order: benzene > toluene > o-xylene. This trend can be attributed to the fact that the diffusion resistance encountered by adsorbate molecules when entering the pores increases with the increase in molecular size, which aligns with the simulation results. The slope of the initial segment of the adsorption isotherm curve indicates that the filling rate of adsorbate molecules decreases with increasing molecular size. In multicomponent co-adsorption systems, molecules with faster filling rates preferentially occupy adsorption sites, thereby influencing the adsorption behavior of other components. From this perspective, benzene may exert a greater influence on toluene adsorption than o-xylene does. The filling rate and filling density of NaY for toluene are significantly higher than those of 13X and MCM-41, indicating its superior toluene adsorption performance. Under low partial pressure, toluene adsorption is primarily driven by strong interactions between adsorbate molecules and the pore channels of the adsorbent. Notably, the pore size of NaY is well-matched to the molecular size of toluene, which further enhances these intermolecular interactions. The experimental results were consistent with the simulation predictions at the level of adsorption trends. Both approaches indicate that appropriate pore confinement simultaneously enhances the interaction intensity between the pore and adsorbate molecules while maintaining favorable diffusion accessibility for toluene. This trend-level agreement, rather than a direct numerical match in absolute capacity, confirms the validity of the pore-size-dependent selectivity trend predicted by the simulation. In contrast, MCM-41 with large pore channels weakened confinement effects and reduced adsorption selectivity despite facilitating molecular diffusion. MCM-41 possesses a larger accessible pore volume than the microporous zeolites, yet this does not directly translate into enhanced toluene selectivity. This can be attributed to the weakened confinement effect within its mesoporous channels. These results suggest that, although pore volume affects the available adsorption space, pore-size matching plays a more critical role in regulating selective adsorption behavior.

### 3.4. Dynamic Adsorption Performance of VOCs

**Figure 4:**
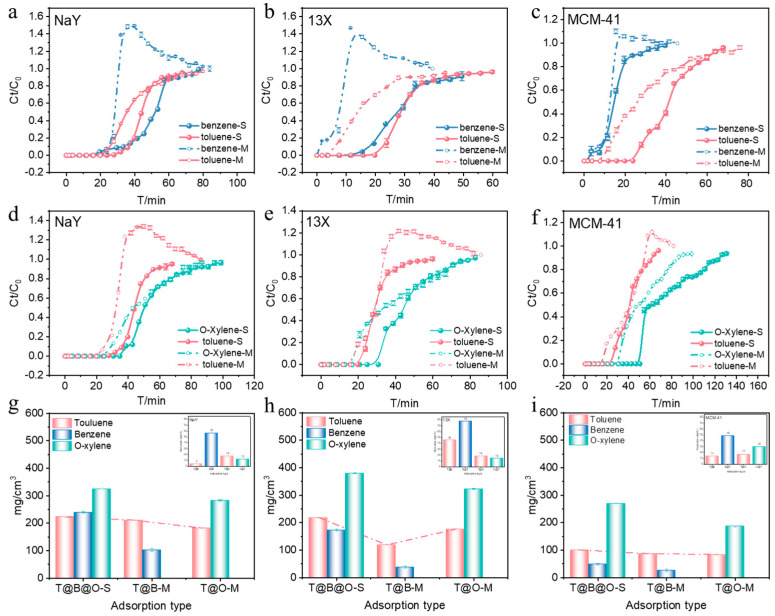
Single-component and binary-component co-adsorption breakthrough curves of toluene and benzene on (**a**) NaY, (**b**) 13X and (**c**) MCM-41, as well as single-component and binary-component co-adsorption breakthrough curves of toluene and o-xylene on (**d**) NaY, (**e**) 13X and (**f**) MCM-41 (solid circles denote single-component adsorption and hollow circles denote binary-component co-adsorption). Adsorption amounts of benzene, toluene and o-xylene per unit pore volume of (**g**) NaY, (**h**) 13X and (**i**) MCM-41 (red: toluene, blue: benzene, green: o-xylene). Error bars represent the standard deviation (SD) of three independent measurements (*n* = 3).

The dynamic adsorption results further corroborate the structure–performance relationship predicted by molecular simulations. In competitive adsorption, selectivity is jointly governed by adsorption affinity, diffusional accessibility, and steric constraints imposed by the pore channels. As shown in Figure 4a–c, on all adsorbents, benzene exhibits an earlier breakthrough than toluene, which is primarily attributable to its smaller kinetic diameter and consequently lower diffusional resistance within the confined pores. Consequently, in the initial stage of binary co-adsorption, benzene molecules preferentially populate the accessible adsorption sites, driven by their faster adsorption kinetics. However, with the progress of adsorption, a pronounced desorption of benzene is observed across all binary systems, suggesting that the initially adsorbed benzene is progressively replaced by toluene. This phenomenon indicates that the adsorption process is governed jointly by diffusion kinetics and thermodynamic adsorption affinity. Although benzene possesses superior diffusion accessibility, the methyl substituent of toluene enhances its interaction with the pore surface through stronger van der Waals forces and local effects, thereby forming a thermodynamically more stable adsorption configuration within the pore [45]. Analogous phenomena were observed in both single-component adsorption and binary-component co-adsorption experiments involving toluene and o-xylene (Figure 4d–f). Specifically, toluene underwent desorption under the competitive effect of o-xylene. Similar competitive adsorption behavior was also observed in the binary adsorption system of toluene and o-xylene In comparison to toluene, o-xylene possesses a larger kinetic diameter and bears two methyl substituents, which strengthen its interaction with the pore wall surface. However, its increased molecular size concurrently induces more pronounced steric hindrance and greater diffusional resistance within the narrow microporous channels. Consequently, the adsorption behavior of o-xylene is governed by the trade-off between enhanced adsorption affinity and restricted diffusional accessibility. Under dynamic adsorption conditions, o-xylene gradually exhibits competitive adsorption behavior towards toluene. Furthermore, the polar methyl groups also help to enhance the adsorption strength of the adsorbate molecules on the polar adsorbent surface. These results indicate that dynamic adsorption behavior is governed by the synergistic effects of confinement-enhanced adsorption interactions and molecular diffusion resistance. Appropriate microporous structures provide stable adsorption configurations for toluene while preserving sufficient transport accessibility, thereby leading to enhanced selective adsorption performance.

**Figure 5:**
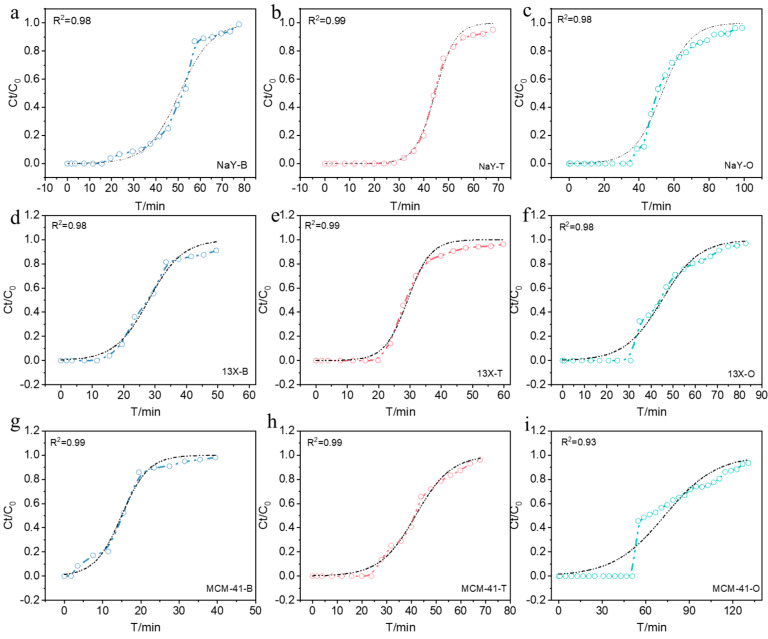
Single-component breakthrough curves of benzene, toluene, and o-xylene on (**a**–**c**) NaY, (**d**–**f**) 13X, and (**g**–**i**) MCM-41. The symbols represent the experimental data, and the dashed black curves correspond to the Y-N model fits.

To quantitatively evaluate the dynamic adsorption behavior of benzene, toluene and o-xylene in different pore structures, the single-component breakthrough curves (Figure 5) were fitted using the Yoon-Nelson (Y-N) model, and the corresponding kinetic constants (K) are summarized in Table S2. The Y-N model adequately described the experimental breakthrough curves, with correlation coefficients (R^2^) exceeding 0.95 for all adsorbents. The relatively high K values for toluene in NaY and 13X indicate that moderate microporous confinement is conducive to the diffusion and adsorption of toluene molecules. In contrast, o-xylene exhibits the lowest diffusion coefficient, as its larger kinetic diameter causes greater steric hindrance within the pore channels. For MCM-41, the K values of benzene, toluene and o-xylene were 0.28, 0.14 and 0.06 min^−1^, respectively. The enlarged mesoporous channels significantly reduced diffusion resistance for adsorbate molecules. However, the weakened confinement effect in mesoporous structures simultaneously reduced the adsorption selectivity toward toluene. These results indicate that selective adsorption is not solely determined by diffusion kinetics; the adsorption stability of adsorbate molecules within the pore channels also governs the competitive adsorption capacity of different molecules for available surface sites.

The adsorption amounts of benzene, toluene, and o-xylene per unit pore volume for the three adsorbents are shown in Figure 4g–i. The adsorption capacities of MCM-41 for the adsorbates are significantly lower than those of NaY and 13X. Both NaY and 13X have the FAU topology, with regular super cage structures and three-dimensional interconnected pore systems, which can ensure rapid molecular diffusion and provide a high density of adsorption sites [46,47]. MCM-41 possesses a one-dimensional ordered mesoporous structure, with pore size substantially larger than the molecular dynamic diameters of benzene, toluene, and o-xylene [48]. The relatively weak intermolecular interactions between molecules and pore walls result in significantly constrained pore volume utilization and adsorption capacity per unit volume. By comparing the adsorption behavior of the same adsorbent towards different adsorbates, the adsorption capacity of o-xylene is markedly higher than that of benzene and toluene on NaY and 13X; this trend is even more pronounced on MCM-41. This is because o-xylene possesses a larger molecular dynamic diameter (approximately 7.4 Å) and a higher molecular polarization. When the pore size is comparable to or slightly larger than the molecular dimensions, stronger interactions and space-filling effects occur, resulting in higher adsorption capacity [47]. While the adsorption capacity of large-molecular-sized adsorbates is limited by spatial steric hindrance, o-xylene exhibits the highest dynamic adsorption capacity under air-disturbed dynamic conditions, because of its strong interactions with the adsorbent’s pore channels.

**Figure 6:**
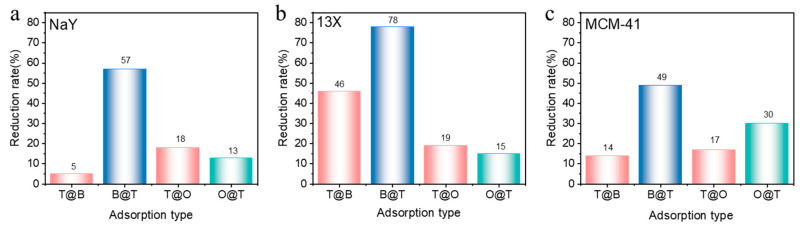
The reduction rate of adsorption capacity in binary-component co-adsorption relative to single-component adsorption, (**a**) NaY, (**b**) 13X, (**c**) MCM-41.

The reduction rate of adsorption capacity in binary-component co-adsorption relative to single-component adsorption was calculated (Figure 6), and this reduction rate ratio was defined as the competitive coefficient of toluene for binary co-adsorption (SI), the smaller the competitive coefficient, the stronger the competitive ability of toluene in binary-component co-adsorption. In the binary-component co-adsorption of toluene and benzene, the toluene competitive coefficients of NaY, 13X, and MCM-41 are 0.09, 0.59, and 0.29, respectively. NaY exhibits the highest adsorption selectivity for toluene, which is consistent with the selectivity coefficients from the simulation results. This indicates that the pore structure of NaY provides the strongest stability for toluene. Despite benzene having a faster adsorption rate, the methyl substituent in toluene enhances its polarity and shortens the distance between the adsorbate molecule and the surface of the pore channel, thereby facilitating the formation of a stable adsorption configuration within the pores and conferring a competitive advantage [49]. In the binary co-adsorption of toluene and o-xylene, the competitive coefficients of toluene for NaY and 13X both exceeded 1, indicating that the adsorption selectivity for o-xylene is higher than that for toluene. Due to its large molecular size and two methyl groups, the o-xylene molecule has a strong interaction with the polar surface of the adsorbent pore channels. However, the toluene adsorption capacities of NaY, 13X, and MCM-41 decreased by 18%, 19%, and 17%, respectively, with no significant differences observed among these values. This is because the steric hindrance effect inhibits o-xylene molecules from occupying the adsorption sites of toluene, thereby exerting a relatively minor impact on the adsorption capacity of toluene.

Dynamic adsorption experiments, integrated with molecular dynamics simulations and static adsorption experiments, further validated the simulation-predicted trend that pore size governs the selective adsorption of benzene, toluene, and o-xylene. The consistency between simulation and experiment in terms of selectivity patterns confirms the reliability of the simulation-guided design strategy. This finding provides a theoretical basis for the precise separation of target adsorbate molecules with similar molecular sizes. Among the studied adsorbents, NaY exhibits the highest adsorption performance and selectivity for toluene.

## 4. Conclusions

In this study, we have systematically investigated the influence of pore size on the competitive adsorption of toluene from binary components by combining molecular dynamics simulations with static and dynamic adsorption experiments. The simulation results reveal that adsorption capacity first increases and then decreases with increasing pore size, a trend arising from the competition between adsorbate–wall interactions and steric hindrance within confined pore channels. More importantly, the simulations identify a pore size of approximately 0.7 nm as optimal for maximizing toluene selectivity, thereby providing concrete design guidelines. This prediction was experimentally validated using NaY, 13X, and MCM-41, confirming that pore architecture is the key structural factor governing selectivity. Beyond these findings, this work establishes pore-size matching as a rational design strategy and provides a validated simulation-experiment framework for predicting multicomponent adsorption performance.

## Data Availability

The original contributions presented in this study are included in the article/Supplementary Material. Further inquiries can be directed to the corresponding authors

## Supplementary Materials

The following supporting information can be downloaded at: https://www.mdpi.com/article/10.3390/ma19143097/s1, Figure S1: Models with different pore sizes; Figure S2: Schematic diagram of the experimental system for the adsorption of single component and the competitive adsorption of binary components; Figure S3: Simulated adsorption isotherms of adsorbent models for (a) benzene, (b) toluene and (c) o-xylene; Figure S4: Infrared spectrum of NaY, 13X, MCM-41; Figure S5: Adsorption isotherms of NaY, 13X and MCM-41 for (a) benzene, (b) toluene and (c) o-xylene at 303 K; Table S1: Pore structure parameters of NaY, 13X and MCM-41; Table S2: K-values for single-component adsorption on NaY, 13X, and MCM-41.Refs [50,51,52] are included in the Supplementary Materials.
