# Supplementary material for: Simulation and Experimental Study of Silica-Based Adsorbents for Competitive Adsorption of Toluene from Binary Components
Source: Materials (Basel). 2026 Jul 19;19(14):3097. doi: 10.3390/ma19143097 (PMC13414452; doi:10.3390/ma19143097)
Supplement: Supplementary file 1 [file materials-19-03097-s001.zip › materials-4417164-supplementary.pdf]

materials

Article

Simulation and Experimental Study of Silica-Based Adsorbents for Competitive Adsorption of Toluene from Binary Components

Yuxin Shi [1,2], Shengzhuo Yuan [1,2], Shiyu Hou [1,*], Gen Huang [1,*], Wanci Shen [2], Feiyu Kang [3] and Zheng-Hong Huang [2,*]

[1] School of Chemical and Environmental Engineering, China University of Mining and Technology (Beijing), Beijing 100083, China; xinxin8640@163.com (Y.S.); ysz1565322666@163.com (S.Y.)
[2] Key Laboratory of Advanced Materials (MOE), State Key Laboratory of New Ceramics and Fine Processing, School of Materials Science and Engineering, Tsinghua University, Beijing 100084, China; shenwc@mail.tsinghua.edu.cn
[3] Tsinghua Shenzhen International Graduate School, Tsinghua University, Shenzhen, 518055, China; fykang@tsinghua.edu.cn
* Correspondence: housy@cumtb.edu.cn (S.H.); huanggen@cumtb.edu.cn (G.H.); zhhuang@mail.tsinghua.edu.cn (Z.-H.H.)

1. Molecular dynamics simulation

1.1. Construction of silicon-based adsorbent models with different pore sizes

To construct the silicon-based adsorbent models, SiO2_21A_3d (amorphous silica) (unit cell parameters: a=b=c= 21.39486 Å, α=β=γ=90°) was selected as the initial unit cell, and a 2×2×1 supercell was built. First, pores with different diameters (0.5 nm, 0.7 nm, 1.0 nm, 1.2 nm, 1.4 nm and 2.0 nm) were created by removing the atoms in the selected area. The number of pores was adjusted under the condition of ensuring the same pore volume of the supercell (Figure S1). Then, the dangling bonds of silicon and oxygen atoms on the pore surfaces were saturated with hydroxyl groups and hydrogen atoms, respectively. Finally, the structure with three hydroxyl groups connected by a silicon atom was selected and replaced with a hydrogen atom. The preliminary models were refined via structural optimization and dynamic relaxation using the DREIDING force field[1], and final adsorbent models were obtained. The cutoff radius for both the Coulombic and van der Waals interactions was set to 12.5 Å. Sequential execution of geometry optimization, annealing, NVT, and NPT dynamics tasks was performed to obtain the adsorbent models with the lowest energy and optimal structure.[2]. During optimization, cell parameters were fixed, and atomic coordinates were adjusted to minimize the total energy of the adsorbent. Periodic boundary conditions were imposed along all three orthogonal directions to replicate the bulk material behavior.

 https://doi.org/10.3390/ma19143097

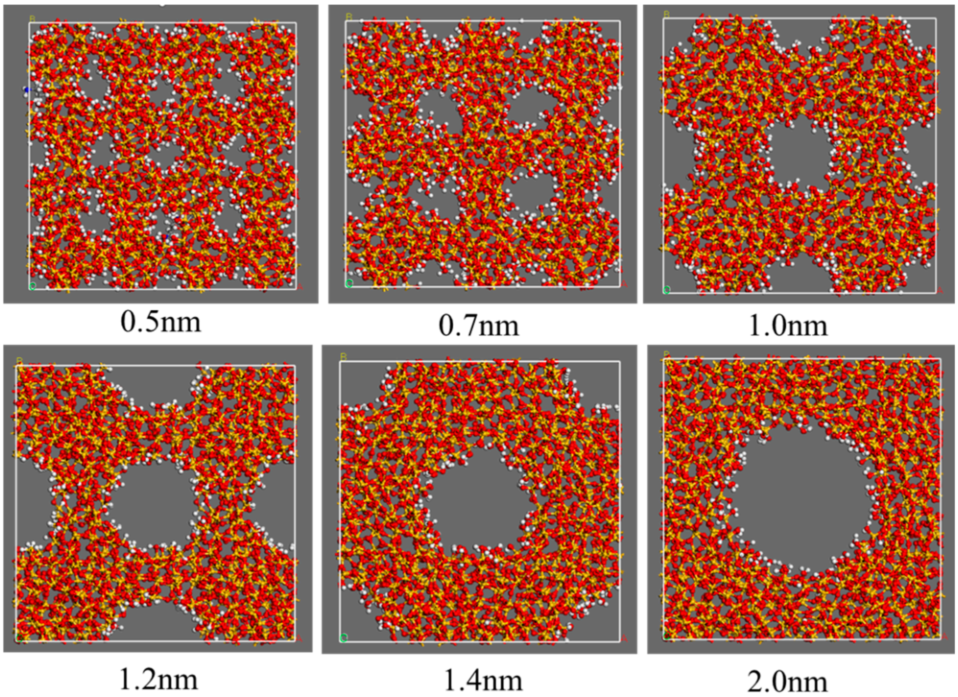

Figure S1. Models with different pore sizes.

1.2. Simulated adsorption isotherms of adsorbate molecules onto adsorbent models

Using the integrated commands in GROMACS, varying quantities of adsorbate molecules were introduced into the bulk region surrounding the adsorbent models. Subsequently, geometrically optimized, annealing, and dynamics tasks were executed on the composite system, adhering to the parameters outlined in Section 1.1.1. The region where the central distance of the adsorbate molecules from the adsorbent surface is less than 1 nm constitutes the adsorption phase, whereas the remaining area represents the bulk phase. The number of molecules within the adsorption phase is quantified utilizing VMD software[3].

The system was first equilibrated for 5×10^6 grand canonical Monte Carlo (GCMC) steps, followed by data collection over an additional 5×10^6 GCMC steps to determine the average adsorbate loading. Equilibration was verified by monitoring the convergence of total potential energy and adsorbate loading; the system was considered equilibrated when these quantities fluctuated around constant mean values without systematic drift[2,3]. The adsorption capacity of the adsorbent models for adsorbate molecules was calculated using the following equation:

$$Adsorption\ capacity = \frac{N}{\mathrm{N_A} V \rho} \tag{S1}$$

Where N is the number of adsorbate molecules adsorbed in the model cell; N_A is Avogadro's constant, which is 6.02×10^{23}; V is the volume of the model cell; and ρ is the density of the model.

1.3. Adsorption selectivity

By combining thermodynamic equations, the ideal gas law, and the partial pressure formula for gases, the IAST theory can predict adsorption selectivity in binary and even multi-component systems. It features a straightforward calculation process, high precision, and excellent agreement with experimental results. The IAST theory finds extensive application in predicting adsorption selectivity for mixed components and evaluating the

performance of adsorbent materials. For adsorption processes involving coexisting binary components, the selective adsorption coefficient is defined as follows: Ai and Aj denote the molar fractions of gases i and j in the adsorbate phase, respectively; Bi and Bj denote the molar fractions of gases i and j in the gas phase, respectively; and S represents the selective adsorption coefficient.

$$S = \frac{Ai/Bi}{Aj/Bj}$$

Specifically, for the adsorption of binary gas components, by applying the partial pressure formula and the law of conservation of mass, it can be readily deduced that:

$$Ai + Aj = 1，Bi + Bj = 1$$

The Ai can be calculated using Wolfram Mathematica software. The calculation code is as follows：

$$FindRoot[qs(i) * \log\left[1 + b(i) * \left(P * \frac{Bi}{Ai}\right)\right] == qs(j) * \log[1 + b(j) * (P * \frac{Bj}{1-Ai})], \{x,0.9999999999\}c]$$

Figure S2

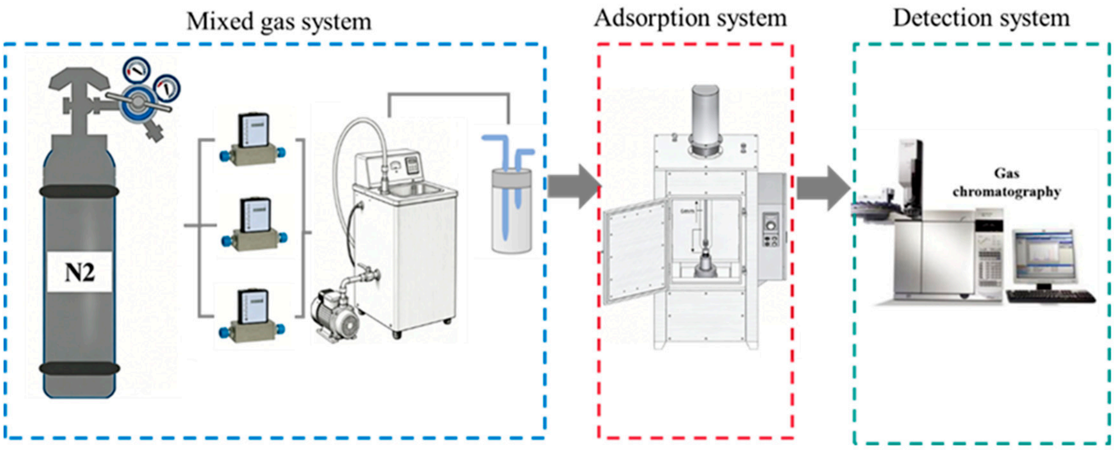

Figure S2. Schematic diagram of the experimental system for the adsorption of a single component and the competitive adsorption of binary components.

Figure S3

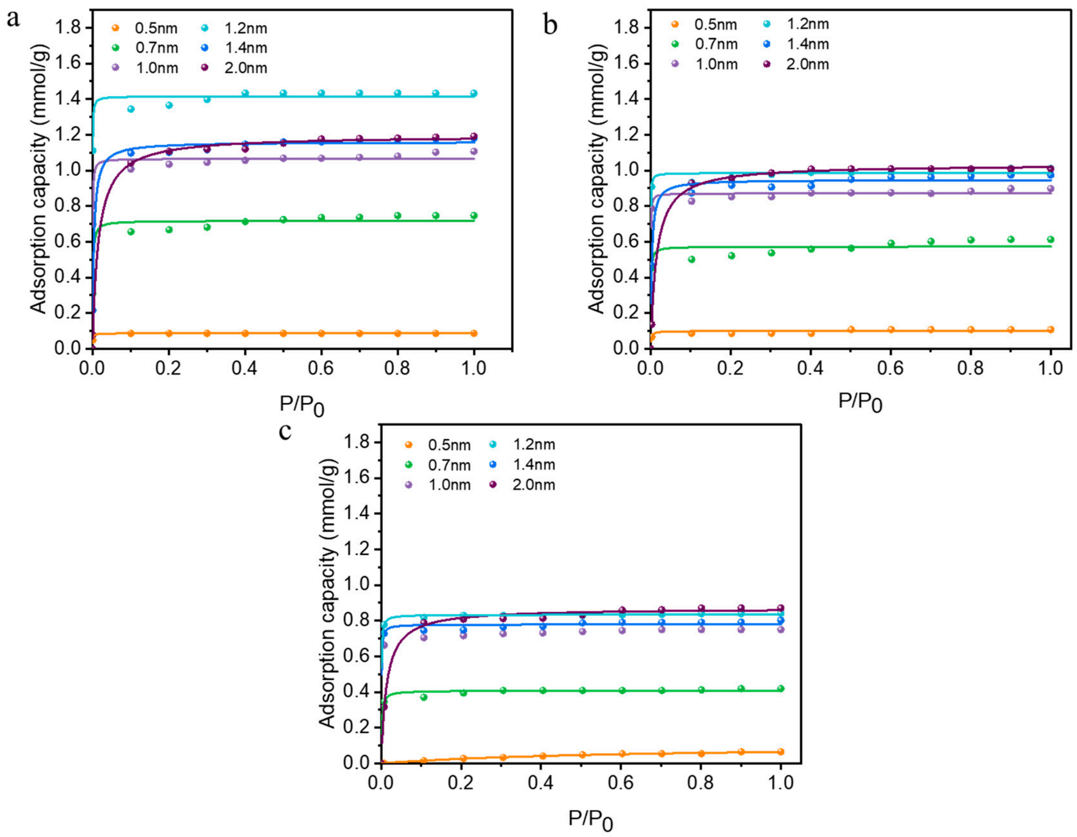

Figure S3. Simulated adsorption isotherms of adsorbent models for (a) benzene, (b) toluene and (c) o-xylene.

Figure S4

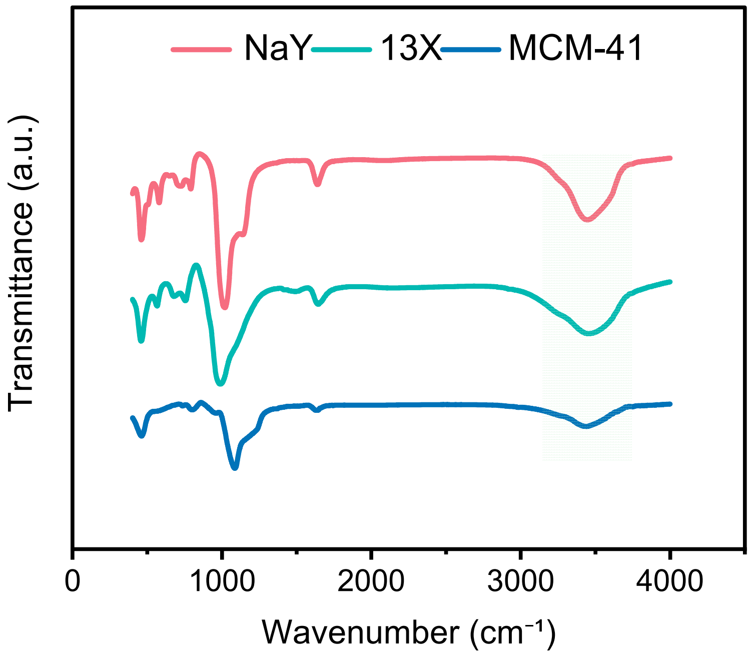

Figure S4. Infrared spectrum of NaY, 13X, MCM-41.

Table S1. Pore structure parameters of NaY, 13X and MCM-41.

Adsorbents	Pore size (nm)	specific surface area (m^2/g)	Pore volume (cm^3/g)
NaY	0.7	605	0.35
13X	1.0	553	0.26
MCM-41	2.2	887	0.65

https://doi.org/10.3390/ma19143097

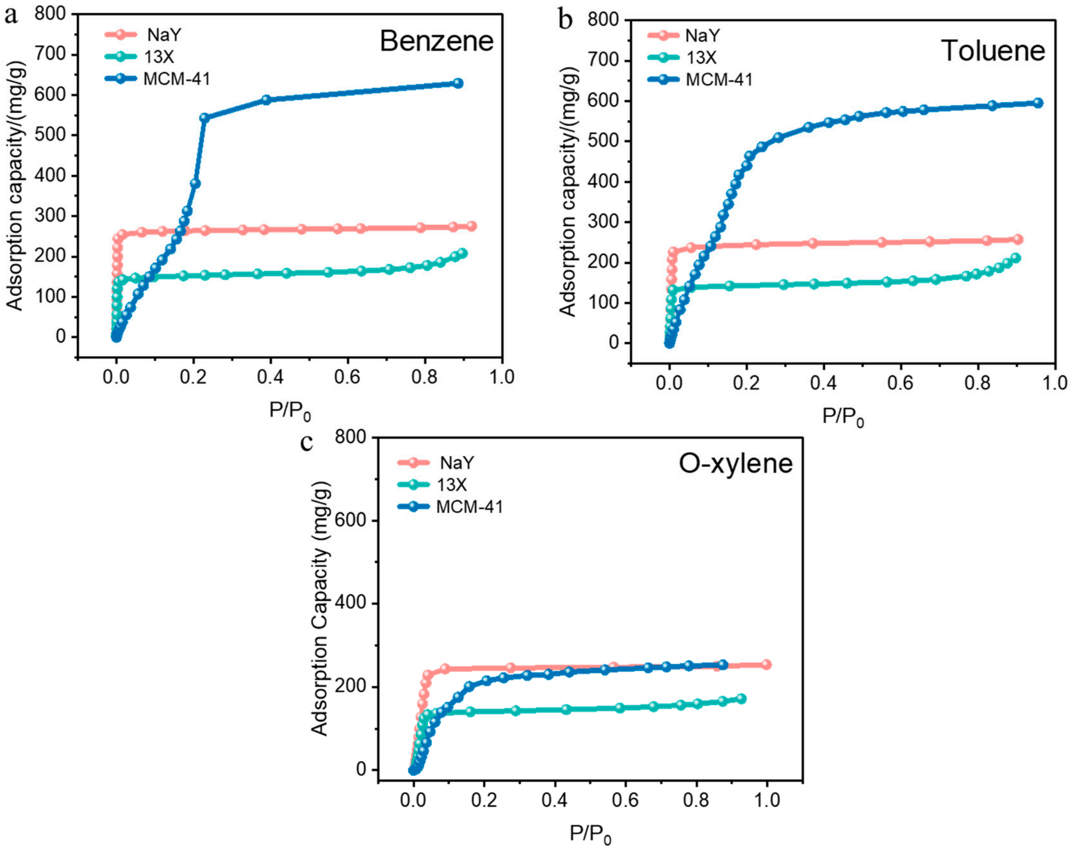

Figure S5. Adsorption isotherms of NaY, 13X and MCM-41 for (a) benzene, (b) toluene and (c) o-xylene at 303 K.

Table S2. K-values for single-component adsorption on NaY, 13X, andMCM-41.

Adsorbents	Benzene	Toluene	O-xylene
NaY	0.16	0.24	0.12
13X	0.18	0.27	0.12
MCM-41	0.28	0.14	0.06

References

S1. Ivanova, E. V., Emelianova, A., Khalizov, A. F. & Gor, G. Y. Molecular Simulation of Benzene Adsorption in Graphitic and Amorphous Carbon Slit Pores. *J. Chem. Eng. Data* **67**, 1765–1778 (2022).

S2. Li, S. *et al.* Molecular simulation of benzene adsorption on different activated carbon under different temperatures. *Microporous and Mesoporous Materials* 302, 110220 (2020).

S3. Elsayed, M. A. *et al.* Insights into the Adsorption of Carbon Dioxide in Zeolites ITQ-29 and 5A Based on Kinetic Measurements and Molecular Simulations. *Nanomaterials* **15**, 1077 (2025).
